# Challenges of quality improvement in the healthcare of South Africa post-apartheid: A critical review

**DOI:** 10.4102/curationis.v42i1.1901

**Published:** 2019-05-29

**Authors:** Winnie T. Maphumulo, Busisiwe R. Bhengu

**Affiliations:** 1 Department of Humanities and Health Sciences, University of KwaZulu-Natal, Durban, South Africa

**Keywords:** challenges in healthcare, healthcare system, lack of resources, quality service delivery, South Africa

## Abstract

**Background:**

There is overwhelming evidence that the quality of health care in South Africa has been compromised by various challenges that impact negatively on healthcare quality. Improvement in quality care means fewer errors, reduced delays in care delivery, improvement in efficiency, increased market share and lower cost. Decline in quality health care has caused the public to lose trust in the healthcare system in South Africa.

**Objectives:**

The purpose of this study was to identify challenges that are being incurred in practice that compromise quality in the healthcare sector, including strategies employed by government to improve the quality of health delivery.

**Method:**

Literature search included the following computer-assisted databases and bibliographies: Medline (Medical Literature Online), EBSCOhost, Cumulative Index to Nursing and Allied Health Literature (CINAHL), Google, Google Scholar and ScienceDirect. Furthermore, websites were used to source policy documents of organisations such as the National Department of Health in South Africa and the World Health Organization.

**Results:**

Seventy-four articles were selected from 1366 retrieved. These articles quantify problems facing quality care delivery and strategies used to improve the healthcare system in South Africa.

**Conclusion:**

The findings revealed that there were many quality improvement programmes that had been initiated, adapted, modified and then tested but did not produce the required level of quality service delivery as desired. As a result, the Government of South Africa has a challenge to ensure that implementation of National Core Standards will deliver the desired health outcomes, because achieving a lasting quality improvement system in health care seems to be an arduous challenge.

## Introduction

Delivery of quality health care is a constitutional obligation in South Africa (Stuckler, Basu & Mckee 2011:165). Government has therefore introduced numerous developments and programmes to improve health care, efficiency, safety and quality of delivery and access for all users (Mogashoa & Pelser 2014:142), and there have been major changes in health policy and legislation to ensure compliance in delivering quality care (Moyakhe 2014:80). Despite a number of commendable goals having been set by government for improved quality of service delivery in healthcare settings, reports by media and communities in 2009 revealed that services in public health institutions were nonetheless failing to meet basic standards of care and patient expectations (National Department of Health 2012:4). This has caused the public to lose trust in the healthcare system (Zubane 2011:1). Koelble and Siddle (2014:1118) describe the healthcare system in South Africa as ruined and in serious need of repair.

Many of problems in the South African healthcare system can be traced back to the apartheid period (1948–1993) in which the healthcare system was highly fragmented, with discriminatory effect, between four different racial groups (black, mixed race, Indian and white) (Baker 2010:79). To worsen the situation, the apartheid government developed 10 Bantustans (the so-called ethnic homelands) into which Africans were unwillingly segregated, and each of which had their own departments of health with their professional bodies (Baker 2010:80). This led to deterioration in health system delivery because of lack of resources, and poor communities were especially affected (Chassin & Loeb 2013:462).

Huge efforts have been made to improve the quality of healthcare delivery in South Africa since 1994 elections, but several issues have been raised by the public regarding public institutions. Among the many, the following seven issues are discussed in this article: prolonged waiting time because of shortage of human resources, adverse events, poor hygiene and poor infection control measures, increased litigation because of avoidable errors, shortage of resources in medicine and equipment and poor record-keeping.

### Prolonged waiting time because of shortage of human resources

A major weakness in sub-Saharan African health systems is inadequate human resources. Africa is said to have less than one health worker per 1000 population compared to 10 per 1000 in Europe (Fonn, Ray & Blaauw 2011:658). Barron and Padarath (2017:4) noted that health problems in South Africa are worsened by unequal distribution of health professionals between the private and public sectors, coupled with unequal distribution of public sector health professionals among the provinces. In a study conducted by Tana (2013:82), participants affirmed the insufficiency and inadequacy of health workers which they described as leading to physical and mental exhaustion, and in some cases to further deterioration of their medical condition.

### Adverse events

Other incidents reported were patients who developed complications, and in some cases died, because they were turned away from the public healthcare facility or denied access to healthcare service. The *Sunday Tribune* (08 March 2015:2) reported on the family of a 35-year-old woman that blamed tertiary hospital staff in KwaZulu-Natal for her death after she was allegedly turned away from the hospital despite being gravely ill. Kama (2017:2) reported the case of a 1-year-old baby who died on his grandmother’s back after they were turned away from three different healthcare facilities in one of the townships in Cape Town. In another incident in the same township, a teenager gave birth on the pavement outside the gates of a health facility because she was not allowed access (Kama 2017:2)

### Poor hygiene and poor infection control measures

According to Young (2016:20), public healthcare facilities exhibit numerous shortcomings such as long waiting times, poor-quality healthcare delivery, old and poorly maintained infrastructure, and poor disease control and prevention practices. According to Dunjwa (2016:1) and the South African Medical Association (2015:36), most facilities had problems such as poor waste management, lack of cleanliness and poor maintenance of grounds and equipment. In a study by Nevhutalu (2016:138), patients and staff confirmed that some departments had an unacceptable physical environment (e.g. dirty toilets) for delivery of quality health care.

### Increased litigation because of avoidable errors

There has been a proliferation of medical negligence litigation against the Department of Health, leading to large payouts which have put further strain on the health budget. At a medico-legal summit in Pretoria (09–10 March 2015), Health Minister Dr Aaron Motsoaledi described these claims as reaching ‘crisis’ level: ‘The nature of the crisis is that our country is experiencing a very sharp increase – actually an explosion in medical malpractice litigation – which is not in keeping with generally known trends of negligence or malpractice’ (Kollapen et al. 2017:3). In a report indicating medico-legal claims paid by government in each province in South Africa (presented at the summit by the acting Chief Litigation Officer of the Department of Justice and Constitutional Development), the total amount paid out for litigation in 2015 was R498 964 916.72; the Department of Health in KwaZulu-Natal led with total claims paid amounting to R153 612 355.49 and with over 5 billion rand in pending claims against the province (Kollapen et al. 2017:16).

The South Africa Nursing Council likewise reported a rise in misconduct cases against nurses, which indicates that the rights of both patients and families were violated (National Department of Health 2013:38). Kukreja, Dodwad and Kukreja (2012:11) further verified the incidence of malpractice litigation claims involving the nursing community, although there have not as yet been any scientific studies conducted in South Africa concerning the nursing community.

### Shortage of resources in medicine and equipment

TimesLIVE (14 June 2018) reported concerns raised by some members of the public regarding the shortage of equipment in hospitals that leads to fatal delays in urgent surgery. Work backlog causes extended delay for some patients awaiting treatment, such as cancer patients who are affected by the lack of oncology doctors and of equipment, and long waiting lists for surgery or diagnosis, also because of the lack of equipment. According to the report, the long waiting times for medical intervention potentially exposed patients to development of complications or even loss of life; public hospitals, in the words of the report, have become ‘a death-trap for the poor’ (TimesLIVE 2018:5). A study by Mokoena (2017:67) revealed about the lack of material resources, equipment and supplies (e.g. glucometers for monitoring blood glucose and needles for lumbar puncture in investigating or diagnosing meningitis), resulting in prolonged patient stay in the hospital. Participants also mentioned that the scan machine was not in proper condition, and that patients were therefore referred to other hospitals for investigations or they had to wait until the machine was fixed, resulting in delayed diagnosis and treatment (Mokoena 2017). Manyisa and Van Aswegen (2017:36) reported that the lack of administrative equipment and skilled professionals adversely affects the quality of care offered in health institutions.

### Poor record-keeping

Kama (2017:80) points out that poor record-keeping causes unnecessary delays for patients. Sometimes, patients’ folders are missing or lost, and instead of healthcare workers explaining this to the patient, they simply let the patient wait (Kama 2017:80). In worst scenarios, the medical history of the patient is lost, which can create further complications leading to incorrect diagnosis and in some cases death of the patient (Kama 2017:80). As reported by the *Mercury* (09 April 2015), the Pietermaritzburg High Court ordered a district hospital in KwaZulu-Natal to hand over medical records to the patient’s attorney in a case where the patient had in July 2006 delivered twins in the hospital, allegedly losing one of the twins while the surviving twin suffered from cerebral palsy because of hospital neglect (Regchand 2015:2).

The objective of this study was to highlight the challenges that are being incurred in practice that compromise quality in the healthcare sector, including strategies employed by government to improve the quality of health delivery.

## Methodology

Articles from 1996 to 2018 were identified from the following online databases: Medline (Medical Literature Online), Ebsco Host, Cumulative Index to Nursing and Allied Health Literature (CINAHL), Google, Google Scholar and ScienceDirect. A comprehensive search was performed to identify 74 articles out of 1366 articles, which account for problems facing quality care delivery and the health care system in South Africa.

**FIGURE 1:**
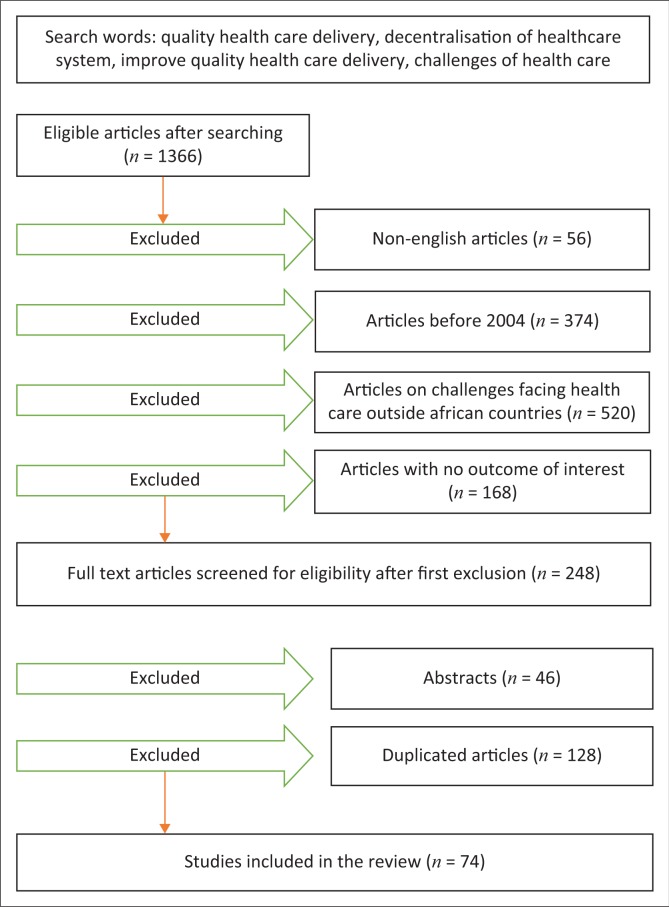
Flow diagram of data collection process and applied inclusion criteria.

Quantitative and qualitative publications were considered for inclusion in the study. The inclusion and exclusion criteria (see Table 1) were applied in the process of data search to acquire the chosen articles. Figure 1 describes data collection process and applied inclusion criteria.

**TABLE 1:** Inclusion and exclusion criteria.

Inclusion criteria	Exclusion criteria
Articles written in English	Non-English articles
Government documents, gazettes, newspapers and press releases	Abstracts were excluded
Articles published from 2004 to date	Articles published before 2004
Articles that are relevant to the topic and question	Articles not relevant to research questions
Articles discussing healthcare problems in Africa	Healthcare problems outside Africa

On reaching consensus about the relevant articles, an annotated bibliography was developed. The annotated bibliography involved summarising and interpreting the articles in the authors’ own words (paraphrasing) for each reference. Common themes of the research topic were extrapolated from the annotations, and the literature was then organised according to these themes and subthemes. The themes were rearranged in chronological order according to the logic of the subject. More intense reading of the articles followed, which culminated in the integration of the emergent themes according to the coherence of the various segments of the literature. Elimination of some initially included articles, and addition of some others, reoccurred at this stage, and some themes were reorganised to fit the logic of the article.

The literature review was then written in relation to each thematic section, using the draft annotations and grouping-related articles either as supporting or as contradicting references. References were cited to support the evidence wherever it was necessary (www.drjayeshpatidar.blogspot.com, retrieved on 27 July 2018).

## Results

### Challenges facing the healthcare system in South Africa

Although South Africa is well known for having a progressive constitution with strong protection of human rights and of the rights of all its citizens to access quality healthcare delivery (Republic of South Africa 1996:13), challenges in delivery of quality health care still exist. Challenges facing the healthcare system in South Africa that are covered in this article are as follows: unequal distribution of resources, management and leadership crisis, increased disease burden, pull and push factors and slow progress in restructuring the healthcare system, including strategies adopted by government to improve the quality of healthcare delivery.

### Unequal distribution of resources

Presently, of the estimated population of 55.5 million (National Department of Health 2016), about 84% of South Africans depend on the public health sector for their healthcare needs (Naidoo 2012:149). Only 16% of South Africans belong to medical aid schemes, and they are attended to by the private sector (Naidoo 2012:149). The Ten Point Plan developed by the National Department of Health (2010–2013) estimated the cost of insured people in the private sector to be 20%, set against 80% for uninsured South Africans in the public sector (National Department of Health 2014:8). The 16% who belong to medical aid schemes consume over 50% of the total healthcare expenditure (National Department of Health 2014:8), while the remaining 84% of the population depend on the under-resourced public sector. In addition, the Department of Health reports that about 80% of medical specialists in South Africa serve the same 16% population in the private sector. However, ECONEX (2013:1) denied that the private sector had more resources serving less population in comparison with the public sector. Some people access the private sector through out-of-pocket expenditure because of the increased demand for quality healthcare services – a need which the public sector is seemingly unable to meet. Another argument is that 63% of general practitioners are working in the public sector, whereas 59% of specialists work in the private sector, some of whom also work part time in the public sector (ECONEX 2013:2). Lastly, according to ECONEX, data revealed that about 62% of nurses employed in the public sector also moonlight in the private sector (ECONEX 2013:2).

Unequal distribution of resources in the healthcare system is also caused by rapid urbanisation in South Africa, estimated currently at 62% of the total population (Turok 2012:8). With health facilities in urban areas having been designed and built to cater for an existing total population (Kon & Lackan 2008:2272; Oladipo 2014:324), the sudden influx of people into cities forces health facilities to function beyond capacity. This has led to overcrowding, and, in turn, to lack of resources and added strain on an already overtaxed healthcare system, because in South Africa, it is unconstitutional to deny anyone access to basic healthcare services, even undocumented immigrants (Mokoele 2012:56).

Shortage of healthcare workers, caused by inadequate production, inadequate recruitment (especially in rural areas), poor retention and staff mismanagement, is a worldwide problem (Veld & Van De Voorde 2014:856). Although health professions in South Africa during the apartheid years were strongly developed through establishment of training centres, serious workforce shortages are recently being experienced (Van Rensburg 2014:3), because of unequal distribution of healthcare workers between the well-resourced private sector and the poorly resourced public sector, and between urban and rural areas (Van Rensburg 2014:3). Shortage of staff is mostly felt at the nursing level because nurses are in the front line of service delivery in health care (Coovadia et al. 2009:821; Voget 2017:16). South Africa’s healthcare system is mainly nurse-based, as acknowledged by the Minister of Health at the 2011 Pretoria National Nursing Summit (05–07 April) in his recognition of the essential role of nurses in achieving ‘A long and a healthy life for all South Africans’ (National Department of Health 2013:8).

A study by Pretorius and Klopper (2012:66), reviewing the workforce profile in critical care, found that in public hospitals and private hospitals in South Africa, respectively, only 72% and 80% of the required nursing staff positions were filled. The health facilities in urban areas were designed and built to cater for a certain number of people. The sudden influx of people into cities forces health facilities to function beyond their intended capacity. This leads to inadequate staffing and overcrowding, which in turn cause a drop in the quality of healthcare delivery in urban hospitals (Kamndaya et al. 2014:581).

In addition to patient influx and a quadruple burden of diseases on the South African health system (Ngomane 2010:26), South Africa is also experiencing a particularly debilitating shortage of professionals and skilled people in the public sector compared to the private sector (Heywood 2014:8). Also exacerbating the shortage of human resources in South Africa has been the closure of many nursing colleges in the late 1990s and an exodus of professionals to work for better income either overseas or in the private sector. Job dissatisfaction also leads to loss of healthcare workers (Heywood 2014:8).

### Pull and push factors

A number of ‘pull and push factors’ have been at play in migration and urbanisation in South Africa (Maharaj 2004:6). The period immediately after the demise of apartheid in 1994 was characterised by an influx of people into cities in reaction to the fall of ‘pass laws’ of apartheid, which controlled the movement of people (Ngomane 2010:11). In addition, South Africa, as a developing country, has been attracting increasing numbers of (often unregistered) immigrants crossing the porous borders for a range of political and economic reasons (Mokoele 2012:17–18). Statistics suggest that there are between half and one million undocumented migrants in South Africa (Baker 2010:81).

### Management and leadership crisis

Health outcome reports in South Africa indicate a complete failure in public sector healthcare delivery, with outcomes worse than that of some lower income countries (Centre for Development and Enterprise 2011:45, Pillay-van Wyk et al. 2016:e642), caused by poor leadership and inadequate management, and reflected in a lack of vision, lack of clear philosophy and poor goal setting (Carney 2009:34; Pillay-van Wyk et al. 2016:e642).

Leadership crises can be traced back to the early days of democracy, following the implementation of government policies to improve living conditions in poor households (Franks 2014:7). One of the tasks of the democratic government was to transform the public service by removing all discriminatory practices and policies in the employment line through implementation of the 1997 Employment Equity Bill (Burger & Jafta 2010:4). The purpose of the public affirmative action policies was to improve the aptitudes of the historically underprivileged (Burger & Jafta 2010:4). Affirmative action policy resulted in loss of institutional memory, and many problems in the healthcare system are associated with the placement of inexperienced managers in senior positions (Coovadia et al. 2009:830; Adejumo & Archibong 2013:2).

The affirmative action in South Africa is reported to have led to poor-quality service delivery because it was characterised by nepotism and affiliation rather than skills and merit (Twala 2014:62). Poor service delivery is also exacerbated by tolerance of misconduct, lack of performance management and monitoring strategies that led to many employees ignoring the law (Siddle 2011:6). A study by Kilonzo and Ikamari (2015:124) concluded, however, that affirmative action opportunities had positive impact on the quality of service delivery and that proper application of affirmative action programmes leads to improvement in the quality of service delivery in public institutions.

In South Africa, most managers are promoted to senior positions because of their length of service in the institution, not because of their skill, and they often apply for promotion because it goes with an increase in salary (Pillay 2010:33). This widens the gap between management team and clinical outcomes (Pillay 2010:33). Lack of accountability, coupled with corruption and misconduct among Department of Health officials (Siddle 2011:6), has also caused the government to fail in fulfilling its constitutional mandate to deliver quality health care. This supports Managa’s (2012:4) findings that the key obstacles in the performance at local government level in South Africa are problems with institutional capacity, high levels of corruption and financial mismanagement and a lack of public participation.

A contrary opinion by Baker (2010:81) contends that the crisis in the healthcare system is more than simply a reflection of corruption and poor domestic governance, and that blame ultimately rests on the structures of apartheid; however, irrespective of the cause, the quality of health care still suffers.

### Increased disease burden

Like every developing country, South Africa faces high burden of disease and seems to be failing to combat it (Kahn 2011:30). The impact of HIV and AIDS in Africa, and in sub-Saharan Africa particularly, has devastated healthcare systems to the extent that they are unable to cope with the demands of high-quality delivery (Naidoo 2012:149). Multiple deficiencies and inadequacies caused by fragmentation of the healthcare system, coupled with racial and socio-economic issues, have led to further proliferation of diseases in South Africa, including HIV and AIDS (Van Rensburg 2014:15).

South Africa currently faces a multiple burden of disease, with the HIV and AIDS epidemic coinciding with high burden of tuberculosis, high maternal and child mortality, high levels of violence and injuries and a growing burden of non-communicable diseases (e.g. cardiovascular diseases, diabetes, chronic respiratory conditions and cancer) (Mayosi et al. 2012:2030; Pillay-van Wyk et al. 2016:e644).

Another major cause of morbidity and mortality in the public sector in South Africa is healthcare-associated infections (HAIs) (Dramowski & Whitelaw 2017:192). Approximately one in seven patients entering South African hospitals is at risk of acquiring an HAI because of poor infection prevention and control measures, such as poor waste management and poor handwashing techniques (Dramowski & Whitelaw 2017:193). Other causes of HAI include overcrowding in hospitals, high patient-to-staff ratios, lack of isolation facilities, ageing infrastructure, inadequate environmental cleaning, inter-hospital transfer of patients with drug-resistant infections and inadequate disinfection of medical equipment (Dramowski & Whitelaw 2017:194). Effects of HAIs include lengthened hospital stay, increase in health care cost for already limited financial resources and in some cases death of patients (Dramowski & Whitelaw 2017:193).

### Slow progress in restructuring the healthcare system

Although in the democratic era, equity is at the top of the South African government’s agenda, little has been done to re-allocate resources from private to public health sector (Heywood 2014:8), which has resulted in delays in the implementation of the National Health Insurance (NHI) policy (Toyana & Auriacombe 2013). There is a particularly crucial need in South Africa for government to improve infrastructure in rural communities, where some primary healthcare centres even lack piped water – a clear sign that the public health system is overburdened and incapable of providing consistent quality care (Heywood 2014:8).

### Increase in consumer demands

Rising customer expectations because of increased use of Internet widen the gap between patient expectations and healthcare worker perceptions (Tonsaker, Bartlett & Atrpkov 2014:407). This makes patient care more complex, with unavoidable demand for high-quality care delivery, while resource shrinkage continues (Lateef 2011:163). All issues identified indicate low levels of service delivery quality in public health facilities, threatening the health and lives of all patients and adding cost to the healthcare system (Cullinan 2006:19).

### Strategies adopted by government to improve quality of healthcare delivery in South Africa

The change to a democratically elected government in South Africa in 1994 brought with it a push for change in the health care system, signalled by a number of policy documents. The first step that was taken by the democratic government was decentralisation of the health care system.

#### Decentralisation of healthcare system

South Africa, like other developing countries, has adopted a process of decentralisation in restructuring health care services (Hendricks et al. 2014:60; McIntyre & Klugman 2003:109). The health care system in South Africa is organised into three levels: national, provincial and local government (Winchester & King 2018:202; McIntyre & Klugman 2003:109). Reviewed literature on the benefits and challenges of decentralisation, especially in the health sector, shows ambivalent findings (Alves, Peralta & Perelman 2013:75). Several studies revealed that decentralisation has produced positive effects in developing countries (Alves et al. 2013:76). In these instances, decentralisation strengthened the capacity of local organisations to negotiate with central government structures for increased resource allocation to previously neglected groups (Alves et al. 2013:76). However, other authors believe that decentralisation has intensified problems of disparity in vulnerable populations, leading to poor-quality health care delivery (Regmi et al. 2010:407; Whittaker et al. 2000:207). According to Surender (2014:18), separating policy determinants from policy implementers in South Africa has led to a crisis in health delivery. Policy implementers failed to restrict health funds at provincial level, which has led to health funds being re-directed to other spending based on political priorities (Surender 2014:18).

#### Policies and legislation

One such notable piece of legislation is the Constitution of the Republic of South Africa 1996, approved by the Constitutional Court on 04 February 1997, and the supreme law of the Republic. The Constitution spells out the rights and duties of its citizens and describes the structure of the government. Emerging from the provisions of the Constitution is the Patient’s Rights Charter, which set a common standard for achieving awareness of these rights (Nevhutalu 2016:79). The National Department of Health leads public health in South Africa and is responsible for overall health policy and coordination, deriving its mandate from the Constitution and the *National Health Act* (No. 61 of 2003), as amended. Among other priority programmes and policies introduced by the democratic government include free-health policies and the district-based primary health care system (Van Rensburg 2014:6).

#### Programmes developed to evaluate healthcare delivery

Various approaches have been developed in South Africa to monitor quality health care delivery. One notable approach has been the development of accreditation as initiated by Dr Whittaker in the pilot Accreditation Programme for South Africa launched in 1994 at the University of Stellenbosch. This research project revealed that many institutions did not comply with minimum standards, calling for new emphasis on continuous quality improvement (Whittaker et al. 2011:64). This led to the establishment of the Council for Health Service Accreditation of South Africa (COHSASA) in October 1995, operating as an independent, non-profit organisation (Whittaker et al. 2011:60). COHSASA is organised as a national cooperative effort involving consumers, state and private organisations and health care providers and is the only body implementing accreditation in South Africa.

The National Department of Health has shown strong commitment to improve the quality of health care delivery in public settings (Whittaker et al. 2011:60). Evidence of this commitment was the development of the Ten Point Plan Strategic Framework, as outlined in the Hospital Revitalisation Programme, which pursued improvement of hospital infrastructure, health technology, administrative management and quality service (National Department of Health 2011:7). As previously noted, there has also been the Negotiated Service Delivery Agreement signed by the Minister of Health, which is intended to ensure effective health care delivery for improving health outcomes and strengthening the health system for all South Africans (National Department of Health 2012:5). To this effect, the Office of Health Standards Compliance (OHSC) was established by government in 2013 (National Department of Health 2013:4) to introduce a quality assurance mechanism which will regulate the quality of health services according to a set of norms and standards prescribed by the *National Health Amendment Act* (No. 12 of 2013). The aim of accreditation is to provide confidence to end users that health service providers are, in fact, competent to provide service (ECONEX 2010:6).

The National Core Standards (NCS) tool developed by OHSC is organised into seven cross-cutting domains (National Department of Health 2012:6). The first three are domains involved directly with the core health system business of delivering quality health care: patient rights and safety, clinical governance and care, and clinical support services. The remaining four domains are the support system that ensures that the system is delivering its core business: leadership and corporate governance, operational management, public health, and facilities and infrastructure. Within each domain are sub-domains that are further divided into subsections or critical areas (National Department of Health 2012:6).

In calling on leadership in health care establishments to facilitate inventiveness and change in practice, the National Department of Health promotes application of NCS as a benchmarking tool for quality of care (Lourens 2012:3). The NCS are to be used as a guide for managers at all levels, indicating the expected service delivery and how to plan for quality care delivery. The NCS tool is also used to assess the quality of health care delivery in health establishments in preparation for the introduction of the NHI. Although implementation of NHI is an honourable attempt to address the inequalities in health care delivery system (National Department of Health 2012:6), it has met with much opposition, causing long delay in its ultimate imple-mentation (Toyana & Auriacombe 2013).

## Conclusion

Although much has been done over many years to restructure the health care system and to improve the quality of care being rendered to users, the literature reveals that millions of people in South Africa still suffer preventable harm every day. Medical litigation has dramatically increased both in frequency and in the size of the damages (Malherbe 2012:83). Therefore, still much needs to be done by government, and society at large, to address the issues of poor-quality service delivery. The literature also reveals that the drive to improve the quality of health care in South Africa has not been lacking in interventions or in powerful ideas. It seems, however, that corruption and lack of leadership skills continue to cause long delay in the achievement of quality health care delivery (Siddle 2011:6).

Nevertheless, South Africa has the potential to draw on its experiences of health inequalities and of the detrimental consequences of historical segregation to build high-quality service delivery for the benefit of all its citizens. Particular suggestions made by authors on how to improve the quality of health care delivery in South Africa are the following:

The South African Medical Association (2015:42) agrees that the current physical state of public facilities is disgraceful and not favourable to the delivery of quality health services. Decentralisation must therefore be implemented cautiously, after confirmation that there is sufficient managerial capacity at district level, and senior officials must be held accountable when they fail to deliver quality as required by their job description.

Sithole and Mathonsi (2015:25) contend that for local government to deliver on its constitutional mandate, government needs to strengthen human and material resource in terms of quantity and quality. Government must also commit to root out nepotism and corruption in areas such as recruitment for positions and awarding of tenders for services (South African Medical Association 2015:43).

Although the OHSC is said to be accrediting all hospitals in South Africa, questions still remain as to its objectivity as the accreditation body because it is fully funded by the government (ECONEX 2010). According to the Standards Council of Canada, accreditation bodies need to perform their work independently (ECONEX 2010:6) because the primary purpose of accreditation is to eradicate biased assessment.

According to Tana (2013:80), the Government of South Africa seems to be unable to deliver the quality of health care as promised. It cannot claim to be providing quality health care service to all patients, while patients remain displeased with health care service delivery. This is in line with the findings of Ned, Cloete and Mji (2017:311) who declared that South Africa’s health outcomes remain below what is anticipated from the current health expenditure, although there were some positive changes mentioned in the 2015 South African Health Review. Further research is therefore needed to assess the efficiency of the strategies used to evaluate health care outcomes.
